# Development and evaluation of uncertainty quantifying machine learning models to predict piperacillin plasma concentrations in critically ill patients

**DOI:** 10.1186/s12911-022-01970-y

**Published:** 2022-08-25

**Authors:** Jarne Verhaeghe, Sofie A. M. Dhaese, Thomas De Corte, David Vander Mijnsbrugge, Heleen Aardema, Jan G. Zijlstra, Alain G. Verstraete, Veronique Stove, Pieter Colin, Femke Ongenae, Jan J. De Waele, Sofie Van Hoecke

**Affiliations:** 1 IDLab, Department of Information Technology, Ghent University - imec, Ghent, Belgium; 2 Department of Internal Medicine and Pediatrics, Ghent University, Ghent, Belgium; 3 Department of Critical Care, University Medical Center Groningen, Groningen, The Netherlands; 4 Department of Diagnostic Sciences, Ghent University, Ghent, Belgium; 5 Department of Anesthesiology, University Medical Center Groningen, Groningen, The Netherlands; 6 Department of Critical Care Medicine, Ghent University Hospital, Ghent, Belgium

**Keywords:** Critically ill, Intensive care, Machine learning, Piperacillin/tazobactam, Population pharmacokinetics, Therapeutic drug monitoring, Uncertainty quantification

## Abstract

**Background:**

Beta-lactam antimicrobial concentrations are frequently suboptimal in critically ill patients. Population pharmacokinetic (PopPK) modeling is the golden standard to predict drug concentrations. However, currently available PopPK models often lack predictive accuracy, making them less suited to guide dosing regimen adaptations. Furthermore, many currently developed models for clinical applications often lack uncertainty quantification. We, therefore, aimed to develop machine learning (ML) models for the prediction of piperacillin plasma concentrations while also providing uncertainty quantification with the aim of clinical practice.

**Methods:**

Blood samples for piperacillin analysis were prospectively collected from critically ill patients receiving continuous infusion of piperacillin/tazobactam. Interpretable ML models for the prediction of piperacillin concentrations were designed using CatBoost and Gaussian processes. Distribution-based Uncertainty Quantification was added to the CatBoost model using a proposed Quantile Ensemble method, useable for any model optimizing a quantile function. These models are subsequently evaluated using the distribution coverage error, a proposed interpretable uncertainty quantification calibration metric. Development and internal evaluation of the ML models were performed on the Ghent University Hospital database (752 piperacillin concentrations from 282 patients). Ensuing, ML models were compared with a published PopPK model on a database from the University Medical Centre of Groningen where a different dosing regimen is used (46 piperacillin concentrations from 15 patients.).

**Results:**

The best performing model was the Catboost model with an RMSE and $R^2$ of 31.94–0.64 and 33.53–0.60 for internal evaluation with and without previous concentration. Furthermore, the results prove the added value of the proposed Quantile Ensemble model in providing clinically useful individualized uncertainty predictions and show the limits of homoscedastic methods like Gaussian Processes in clinical applications.

**Conclusions:**

Our results show that ML models can consistently estimate piperacillin concentrations with acceptable and high predictive accuracy when identical dosing regimens as in the training data are used while providing highly relevant uncertainty predictions. However, generalization capabilities to other dosing schemes are limited. Notwithstanding, incorporating ML models in therapeutic drug monitoring programs seems definitely promising and the current work provides a basis for validating the model in clinical practice.

## Background

### Introduction

The morbidity, mortality, and healthcare costs associated with infectious diseases in the Intensive Care Unit (ICU) continue to be a major health issue [1]. Antimicrobial therapy remains the mainstay of treatment, with piperacillin/tazobactam (TZP) being one of the most frequently prescribed antimicrobials in the ICU worldwide [2].

Achieving therapeutic antimicrobial concentrations likely improves the clinical outcome, avoids drug toxicity, and reduces the burden of antimicrobial resistance [3, 4]. In the past few years, a wealth of evidence emerged demonstrating possible suboptimal and difficult to predict beta-lactam antimicrobial concentrations in critically ill patients following standard dosing regimens [5]. Several experts have recommended abandoning this ‘one-size-fits-all approach’ in ICU patients and have moved towards individualized antimicrobial dosing to reach therapeutic windows[6, 7]. This decision is based on early studies indicating that individualized dosing may decrease mortality in ICU patients [3, 8, 9]. An alternative would be to dose up to the point of toxicity to ensure target attainment is reached. For TZP, dose modifications and dosing interval adjustments are usually performed according to the renal function. A renal dysfunction suggests dose reduction and a very good renal function suggests a longer dosing administration time and/or higher dosing [3, 8, 10].

An obstacle limiting the implementation of more advanced individualized therapy is the absence of readily available measured beta-lactam antimicrobial concentrations in daily routine [3, 11]. Measuring plasma concentrations of beta-lactam antimicrobials can be performed using therapeutic drug monitoring (TDM) [12]. However, TDM is not routinely performed as the means and expertise for TDM are not always available and the time interval between sampling and availability of results is often long. An alternative to measuring the concentration is predicting it. Predicting plasma concentrations of beta-lactam antimicrobials is possible with Population pharmacokinetic (PopPK) analysis. PopPK analysis uses non-linear mixed effect modeling to simulate the relationship between the antimicrobial concentrations, the dose, time, and the patient-specific covariates [13]. Once a PopPK model is developed, a typical Pharmacokinetic (PK) profile can be generated and antimicrobial concentrations can be predicted for a given patient [13]. However, PopPK models are frequently based on (small) retrospective datasets from studies that were not primarily aimed towards the development of a PopPK model. This results in context-/subgroup-specific models with poor extrapolation properties to other datasets [14]. Therefore, sample size calculations and simulations to optimize the experimental design for PopPK modeling are not always performed as they require a considerable amount of computational resources [15]. Hence, many of the current beta-lactam PopPK models in ICU patients often have poor predictive accuracy. As a result, dosing recommendations based on these PopPK models are context-specific and vary substantially from one model to another [16, 17]. To overcome these limitations of small, context- and subpopulation-specific PopPK models, large pharmacokinetic data-sharing initiatives are currently underway [18].

Another strategy to overcome these PopPK measuring and prediction limitations is to predict antimicrobial concentrations with machine learning (ML) models [19]. ML uses algorithms to find patterns and relationships in data and is not dependent on many underlying domain-specific assumptions [20]. Therefore, ML models could find new relationships between antimicrobial concentrations and covariates and use much more covariates compared to PopPK models.

However, conventional ML predictions often only provide single outputs without any information about this prediction. This all-or-nothing output often limits model acceptance and inhibits risk assessments in clinical practice [21], especially in high-risk environments such as the ICU. It is possible to increase the trust and understanding in these models by providing extra prediction information using uncertainty quantification, which is especially useful for making decisions.

Hence, in this paper, we used three regression ML models to predict total plasma concentrations of piperacillin in critically ill patients. These models will then be compared to a developed and published PopPK model to research the added value of ML with respect to PopPK on an internal and external dataset. Additionally, a general distribution-based uncertainty quantification framework, the quantile ensemble, is proposed to provide uncertainty estimates for the predicted concentrations using the final ML models. At last, we also propose two uncertainty quantification performance metrics, the Absolute Distribution Coverage Error (ADCE) and the DCE (Distribution Coverage Error), usable in model selection and uncertainty quantification performance evaluation.

### Related work

#### Drug concentration prediction

Earlier works already explored the idea of using various ML models, such as support vector machines, gradient boosting trees, XGBoost, and neural networks, to predict drug concentrations for tacrolimus, remifentanil, gentamicin, risperidone, teicoplanin, phenytoin, and warfarin [22–29]. A recent study explained and validated the predictions of teicoplanin trough concentrations using Shapley values while combining the best models into a single ensemble [28, 30]. Another study used XGBoost to act as a classifier, trained on virtual patients, to select the best PopPK model to aid TDM-guided dosing [31]. However, none explored the prediction of piperacillin plasma concentrations directly using machine learning models nor did they include uncertainty quantification for the concentration prediction while comparing to a published PopPK model on an internal and external evaluation dataset.

#### Regression uncertainty quantification

In regression problems the observable targets *y* are theorized to consist of a ground truth function *f*(*x*), given the input features *x*, and additive noise $\epsilon$. When predicting the target variable, we try to find an estimator such that $\hat{y}=\hat{f}(x)$ closely resembles the target *y*. In contrast, in uncertainty quantification, the goal is to correctly approximate and describe the predictive distribution $P_{\hat{{\textbf {Y}}}}$ of the outputs $\hat{y}$ such that it correctly encompasses all sources of uncertainty [32].

Two metrics are important for uncertainty quantification evaluation: calibration and sharpness. The calibration quantifies how well the predictive distribution captures the ground truth uncertainty of the predictions by evaluating and comparing every quantile of the predictive distribution. The sharpness indicates the size of this predictive distribution, which is in this case the standard deviation $\sigma$. The sharpness and calibration are both required to effectively evaluate the predictive distribution, and a trade-off exists between these two metrics [33]. The goal is to have a predictive distribution that is as small as possible but still has perfect calibration. Zhao et al. discussed various theoretical requirements for regression distribution-based uncertainty quantification such as different kinds of calibration and sharpness [33].

Various solutions for distribution-based uncertainty quantification for regression problems have already been proposed such as mean-variance methods and Bayesian-based models. Mean-variance methods output a mean and variance of the Gaussian distribution by optimizing the negative log-likelihood loss. More advanced solutions use Bayesian frameworks to output a Gaussian distribution by e.g. directly optimizing the Kullback-Leibner divergence, such as Bayesian Neural Networks. These methods are often only bound to neural networks or specific models and therefore not model-agnostic [32].

## Methods

### Data

#### Ghent university hospital patients

Data from patients, included in a prospective observational study conducted between March 2016 and April 2018 in the surgical ICU of the Department of Critical Care of Ghent University Hospital (GUH, Ghent, Belgium), a tertiary university hospital with 52 ICU beds, were used. Ethical approval was obtained from the Ghent University Hospital Ethics Committee (registration number 2016/0264). Patient agreement was obtained via opting out before participation. Patients admitted to the surgical ICU and receiving both targeted and empirical piperacillin/tazobactam (4g/0.5 g powder for solution for infusion; Fresenius Kabi n.v., Schelle, Belgium) for at least 24 h in continuous infusion were screened for eligibility. Patients younger than 18 years and patients receiving extracorporeal membrane oxygenation or renal replacement therapy (RRT) during antimicrobial therapy were excluded.

Piperacillin antimicrobial concentrations and additional data, such as biochemistry, demographic data, the Sequential Organ Failure Assessment score (SOFA) on the day of sampling [34], and the Acute Physiology and Chronic Health Evaluation (APACHE II) score on admission, were prospectively collected. Biochemical variables such as serum creatinine, albumin, platelets, lactate, white blood cells, and bilirubin were determined from the same drawn blood sample as the antibiotic plasma concentration. Creatinine clearance ($CL_{cr}$) was determined by measuring urinary creatinine concentrations from an 8-h urinary collection (m$CL_{cr}$). If no m$CL_{cr}$ was available, estimated glomerular filtration rate (eGFR) as calculated by the CKD-EPI equation was used [35]. TZP dosing in GUH was as follows: loading dose of 4/0.5 g/30 min immediately followed by a continuous TZP infusion depending on m$CL_{cr}$ (eGFR): m$CL_{cr}$ (eGFR) < 15 mL/min: 8/1 g/24 h, m$CL_{cr}$ (eGFR) 15–29 mL/min: 12/1.5 g/24 h and for a m$CL_{cr}$ (eGFR) $\ge$ 30 mL/min 16/2g/24h. Measured piperacillin concentrations were not disclosed to the treating physicians.

Remnants of the blood gas syringes (RAPIDLyte; Siemens Healthcare Diagnostics, Deer- field, IL) taken as part of the routine arterial or venous blood sample every morning at 6 a.m. were collected as steady-state study material. Samples were centrifuged and plasma was frozen at -80C awaiting batch analysis. Total plasma concentrations of TZP were analyzed by the Laboratory of Clinical Toxicology and Drugs analysis of the Department of Laboratory Medicine of GUH using a validated fast ultra-performance liquid chromatographic method with tandem mass spectrometric detection (UPLC-MS/MS) [36].

The UPLC-MS/MS system consisted of a Waters Acquity UPLC instrument coupled to a TQD triple-quadrupole mass spectrometer (Waters Corp., Milford, MA). Separations were performed on an Acquity UPLC BEH C18 column (100 mm $\times$ 2.1 mm, 1.7 $\upmu$m particle size ) equipped with a 0.2 $\upmu$m precolumn filter unit and a guard column (Waters Corp., Milford, MA). Analytes were measured in the multiple reaction monitoring (MRM) mode. The flow rate was set at 0.4 mL/min. The column and autosampler tray temperature were set at 50 $^{\circ }$C and 4 $^{\circ }$C respectively. 40 $\upmu$L of the extract was injected into the column. The MS/MS instrument was operated with a capillary voltage of 1.00 kV, a source temperature of 140 $^{\circ }$C, and desolvation gas (nitrogen) at 400 $^{\circ }$C with a flow of 800 L/h. Analytes were measured in the electrospray positive (ESI+) mode. The deuterated standard D5- piperacillin from Toronto Research Chemicals (Ontario, Canada) was used as an internal standard. Data were acquired using Masslynx 4.1 software and processed using Quanlynx 4.1 software (Waters Corp., Milford, MA).

#### University medical centre of Groningen patients

For external evaluation a dataset of ICU patients receiving continuous infusion TZP enrolled by Aardema et al [37] from the University Medical Centre of Groningen (UMCG) was used. Only UMCG patients fulfilling all inclusion criteria and none of our exclusion criteria were used for evaluation. Some records were duplicates except for the concentration. As these concentrations were close to the other record (±5 mg/L), only the first occurrence was kept [38]. In UMCG, a 24-h urine collection was used to measure $CL_{cr}$. If no such collection was available, the MDRD [39] formula was used to estimate the glomerular filtration rate (eGFR). TZP dosing in UMCG was as follows: loading dose of 4/0.5 g/30 min immediately followed by a continuous TZP infusion depending on m$CL_{cr}$ or eGFR if no urine collection was available: m$CL_{cr}$ (eGFR) < 20 mL/min: 8/1g/24h, m$CL_{cr}$ (eGFR) 20–39 mL/min: 8/1g/24h for the first 24 h, followed by 12/1.5 g/24 h afterward and for a m$CL_{cr}$ (eGFR) $\ge$ 40 mL/min 12/1.5 g/24 h.

#### Data cleaning

Missing values of variables with less than 5% missing were either interpolated or replaced by the previous or next value, depending on data and expert knowledge. Three variables had more than 5% missing values and were handled differently: urine creatinine level, body temperature, and m$CL_{cr}$. Urine creatinine and temperature missing values were replaced with their mean values for the Gaussian process (GP) and multilayer perceptron (MLP) models for numerical stability and with -999 for the Gradient Boosting Tree (GBT) models to indicate missings. Missing m$CL_{cr}$ values were approximated using the adjusted Cockcroft-Gault [40] and MDRD [39] formulas. An optimized weighted sum of these formulas was determined in the cross-validation phase to be $(CockcroftGault+2\cdot MDRD)/3$. The CKD-EPI [35] equation was evaluated but of no additional value. Records still containing missing values after this step were deleted. The number of imputed values per variable can be found in Table 1.

**Table 1:** The number of missing values for all considered features in both datasets with size N

Feature	GUH (N = 752)	UMCG (N = 46)
Albumin (g/dL)	13	7
Bilirubine (mg/dL)	19	20
Creatinin clearance (mL/min)	100	0
Height (cm)	6	0
Hemoglobin (g/dL)	7	36
Lactate (mmol/L)	5	34
Platelets (/mm$^{3}$)	7	20
Serum creatinine (mg/dL)	14	0
SOFA	3	21
Temperature ($^{\circ }$C)	107	N/A
Urine creatinine (mg/dL)	39	12
White blood cells (/mm$^{3}$)	8	36

Features that are not shown in the table contained no missing values

Sequential records of patients were linked with a variable that described the previous concentration of the patient. A default previous concentration of 0 mg/L was used in every first record of a patient to indicate that no known previous concentration was available. A second feature was also included to depict the time to the previous concentration.

#### Study population

After excluding 13 patients with 21 concentrations during data cleaning, 282 patients with 752 piperacillin concentrations were included in the GUH dataset. Patients were split on their patient id into a training set, containing 240 patients and 601 concentrations, and two test sets for model evaluation: a *a priori* test set for evaluating a clinical scenario without a previous piperacillin plasma concentration, and a *a posteriori* test set to mimic the situation where one or more piperacillin plasma concentrations were available. 25 percent of patients with at least two records were used as the test set (same patients for both test sets) for the GUH evaluation. This resulted in a *a priori* test set, containing 42 patients and 151 concentrations, and a *a posteriori* test set with 42 patients and 109 concentrations.

After the exclusion of samples drawn within the first 24 h of TZP therapy and data cleaning, the UMCG dataset consists of 15 patients with 46 concentrations and is used for external evaluation. The UMCG dataset is also split into a *a priori* (15 patients, 46 concentrations) and *a posteriori* test set (12 patients, 31 concentrations). Patient demographics and clinical characteristics for both the GUH and UMCG datasets are shown in Table 2.

**Table 2:** Descriptive statistics for the GUH and UMCG dataset

Variable	GUH (n = 285)	UMCG (n = 15)	*p*-value
*Demographics*			
Sex (male)	183 (64.9%)	13 (87.0%)	0.718
Age, median (IQR) (year)	64 (53–74)	60 (54–66)	0.133
Height, median (IQR) (cm)	170 (165–178)	175 (172–178)	0.101
Weight, median (IQR) (kg)	75.0 (64.2–85.0)	77.0 (70.0–90.0)	0.138
APACHE II score upon admission median (IQR)	23.0 (3.0–29.0)	NA	NA
APACHE IV score upon admission median (IQR)	NA	74.0 (65–87)	NA
SOFA score on the day of sampling median (IQR)	5 (3–8)	12 (9–14)	< 0.001
ICU mortality (%)	33 (11.7%)	4 (26.7%)	0.329
*Admission category (%)*			
Medical	118 (41.8%)	4 (26.7%)	0.599
Surgical	135 (47.9%)	8 (53.3%)	0.883
Trauma	29 (10.3%)	2 (13.3%)	0.662
*TZP treatment*			
Duration of TZP therapy, median (IQR) (days)	3 (1–5)	3 (2–6)	0.373
Piperacillin concentration, median (IQR) (mg/L)	81.0 (54.4–121.4)	50.3 (36.5–80.9)	0.260
No. of blood samples per patient, median (IQR)	2 (1–3)	4 (1–5)	0.350
Timing of blood sample relative to the start of treatment, median (IQR) (hours)	63 (35–115)	30 (12–48)	<0.001
Time to the previous concentration median (IQR) (hours)	24 (24–48)	24 (24–24)	0.023

Variable	GUH (n = 752)	UMCG (n = 46)	*p*-value
*Lab results*			
Serum creatinine, median (IQR) (mg/dL)	0.69 (0.51–1.04)	0.76 (0.52–1.33)	0.255
Measured creatinine clearance, median (IQR) (mL/min)	107.5 (65.5–143.7)	96.9 (40.5–127.9)	0.125
Albumin, median (IQR) (g/L)	23.0 (20.0–26.0)	22.5 (19.0–26.0)	0.257
Platelets, median (IQR) ($10^9$/L)	281.0 (174.0–414.0)	160.0 (123.5–199.2)	0.729
Lactate, median (IQR) (mg/dL)	10.9 (8.5–14.3)	12.0 (9.0–18.0)	0.561
White blood cells, median (IQR) ($10^9$/L)	12.6 (9.9–16.9)	11.7 (8.5–19.5)	0.689
Bilirubin, median (IQR) (mg/dL)	0.60 (0.40–1.10)	0.99 (0.36–2.70)	0.468
Fluid balance in the previous 24 h median (IQR) (ml/24 h)	421.9 (− 359.7 to 1399.3)	816.5 (− 210.0 to 2328.2)	< 0.001

The timing of the lab results is from the first piperacillin concentration available for analysis. *n* is the amount of samples included (patients for demographics, admission category, and TZP treatment and lab samples for lab results

All statistical analyses were performed using Python (version 3.8.5) and NumPy (version 1.19.1). Continuous variables are presented as median with interquartile range (IQR). Categorical variables are presented as counts and percentages (%). For continuous data with a normal distribution, the independent-samples t-test was used to compare means (p-value). In the case of a non-normal distribution, the Wilcoxon rank-sum test was used to compare distributions between groups. For categorical data, Pearson’s $\chi ^2$ or Fisher’s Exact Test were used.

### Machine learning models

#### Models

Three models were selected. Two models were chosen as interpretable models capable of uncertainty quantification to give the clinician insights into the prediction and provide model output confidence: The Quantile regression Gradient Boosting Trees (GBT) (open-source CatBoost library, version 0.25) and Gaussian processes (GP) (GPy library, version 1.9.9). The third model is a Multilayer Perceptron model (MLP) or fully-connected feed-forward neural network (Tensorflow library, Version 2.3.0). The GUH dataset is considered a small dataset, therefore, deep learning approaches are not suitable for this problem. However, to prove this statement, the MLP is included in this study. The MLP model will not be used for uncertainty quantification due to the limited dataset. Therefore, more advanced uncertainty quantifying methods, such as Bayesian Neural Networks, are not considered in this study.

For each ML model, two different sub-models were trained. The first model predicts a concentration when a previous TDM measurement is available and, denoted as the *prev* model, and is used for the a posteriori case. The second model denoted as the *new* model, predicts a concentration when there is no prior TDM measurement available, i.e. a previous concentration of 0, and is used for the a priori case.

For the GP models, the *new* model was built using the Radial Basis Function kernel, while the *prev* model used the Multi-Layer Perceptron kernel to represent the prior knowledge. Each final GBT model is an ensemble consisting of three sub-models: one model dedicated to predicting the concentration, and two models to predict the upper and lower prediction quantile for uncertainty quantification, further referred to as the Quantile Ensemble.

#### Model development strategy

Model development was performed on the GUH training set, using 10-fold cross-validation (CV), where the patients are split using their patient id and the number of measurements per patient was used to stratify the split. The CV was used to select the features, determine parameters, and compare different techniques. Prediction errors were evaluated using the mean error (ME), the mean absolute error (MAE), the root mean square error (RMSE), the coefficient of determination ($R^2$), median absolute percentage error (MdAPE), and median percentage error (MdPE).

For model development, the RMSE was the preferred metric to determine the best model and feature selection algorithms as it quantifies the average error and quadratically penalizes large errors. Furthermore, to determine the best hyperparameters for the Quantile models in the Quantile Ensemble for uncertainty quantification, the proposed Absolute Distribution Coverage Error metric was the preferred metric, minimizing the metric.

#### Feature selection

The features considered for model building were: age (yrs), height (cm), weight (kg), race, sex, SOFA, lactate (mmol/L), serum creatinine (mg/dL), urine creatinine (mmol/L), creatinine clearance ($CL_{cr}$, mL/min), hematocrit (%), platelets (/mm3), white blood cells (/mm3), red blood cells (/mm3), bilirubin (mg/dL), hemoglobin (g/dL), albumin (g/dL), fluid balance (mL/24h), piperacillin/tazobactam (TZP) dose per hour (mg/h), temperature ($^{\circ }$C), AKI stage (cf. KDIGO definition), cumulative administered dose (mg), previous piperacillin concentration (mg/L), reason for ICU admission (i.e. medical, surgical, trauma admission, neurological trauma), dobutamine usage (yes/no), vasopressor usage (yes/no), epinephrine usage (yes/no), dopamine usage (yes/no), norepinephrine usage (yes/no), milrinone usage (yes/no), and phenylephrine (yes/no). These were all available features, collected on the plausibility of prediction impact as judged by clinicians and permission of collection. Adding features to include changes over time did not result in better performances and were therefore not included. The biochemistry features are determined from the same moment of drawing the piperacillin concentration. Together with the other variables, this creates a feature set to predict the concentration at the moment of drawing the blood sample when the biochemistry variables become available. Hence, our model predicts the antimicrobial concentration at the time of drawing the sample using readily available data, without the need of expensive laboratory equipment or the large turnaround time required for the concentration determination.

The feature selection for the GBT ensemble and the MLP model used a novel method called PowerShap, a feature selection algorithm that uses statistical hypothesis testing and power calculations on Shapley values [41]. The GP model is not supported by the PowerShap library for feature selection and therefore forward feature selection was used, iteratively adding features providing the best results. Feature selection was performed before optimizing the hyperparameters and re-executed whenever new techniques, models, or loss functions were tried.

### Uncertainty quantification

Providing uncertainty quantification for any prediction is important, especially for high-risk environments such as the ICU [42]. Therefore, for this study, specific attention was given to providing uncertainty quantification. The goal of the uncertainty quantification in this study is to provide a complete predictive distribution together with the regression output to enable calculating the likelihood that the true drug concentration will be between specific bounds. This is especially useful for evaluating whether the predicted concentration attains a therapeutic drug concentration window.

For the current application, the predictive distribution is assumed to be Gaussian, which is characterized by two parameters: the mean $\mu$, corresponding with the regression output, and the standard deviation $\sigma$. The Gaussian assumption is inherently incorporated into the Gaussian process model. For the other method, the proposed Quantile Ensemble, the assumption is used to provide a predictive distribution.

#### Gaussian process

The output of a Gaussian process is a Gaussian Distribution with estimated mean $\tilde{\mu }$ and estimated standard deviation $\tilde{\sigma }$ and therefore requires no further calculations. Furthermore, the Gaussian process is a homoscedastic Bayesian-based uncertainty quantification method, where the standard deviation is approximately equal for all samples providing a global uncertainty prediction.

#### Quantile ensemble model

There are three models in the Quantile Ensemble Model. One for the regression output or $\tilde{\mu }$ and two for estimating an upper $\tilde{y}_{U}$ and lower quantile $\tilde{y}_{L}$ of the predictive distribution.

First, a specific coverage *p* is defined, which is a hyperparameter that specifies the upper and lower quantile:

1 $$\begin{aligned} Q_{up}= & {} 0.5+\frac{p}{2} \end{aligned}$$

2 $$\begin{aligned} Q_{low}= & {} 0.5-\frac{p}{2} \end{aligned}$$

Then, given the quantile function of the Gaussian distribution for a coverage p:

3 $$\begin{aligned} Q(p;\mu ,\sigma )=\mu +\sigma \sqrt{2}erf^{-1}(2p-1) \end{aligned}$$

With $\mu$ and $\sigma$ parameters of the Gaussian distribution. It is then possible to derive $\tilde{\sigma }$ using the predicted upper $\tilde{y}_{U}$ and lower $\tilde{y}_{L}$ quantiles:

4 $$\begin{aligned} \tilde{\sigma }= \frac{\tilde{y}_{U}-\tilde{y}_{L}}{\sqrt{2}\cdot erf^{-1}(p)} \end{aligned}$$

#### Distribution inferences

Given the estimated distribution parameters $\tilde{\mu }$ and $\tilde{\sigma }$ and the Gaussian distribution quantile function, any prediction interval can now be estimated for a given coverage *p*:

5 $$\begin{aligned} \begin{aligned}{}[\tilde{y}_{L_p},\tilde{y}_{U_p}]=\left[ Q\left( \frac{1-p}{2},\tilde{\sigma },\tilde{\mu }\right) , Q\left( \frac{1+p}{2},\tilde{\sigma },\tilde{\mu } \right) \right] \end{aligned} \end{aligned}$$

Additionally, the estimated coverage percentage $\tilde{p}$ between any upper $y_{U}$ and lower bound $y_{L}$ can then be calculated as follows:

6 $$\begin{aligned} \tilde{p}=erf\left( \frac{y_{U}-\tilde{\mu }}{\tilde{\sigma }\sqrt{2}}\right) -erf\left( \frac{y_{L}-\tilde{\mu }}{\tilde{\sigma }\sqrt{2}}\right) \end{aligned}$$

By predicting the quantiles and recalculating the predictive distribution for each individual sample, the Quantile Ensemble becomes a heteroscedastic method. In contrast to a homoscedastic model, a heteroscedastic model provides standard deviations that can differ for each sample, thereby providing an individualized uncertainty prediction. Do note that the provided Quantile Ensemble method can be applied to any model optimizing a quantile loss function, and is not limited to the CatBoost model. Although only two models are required to estimate the distribution, using three models provides higher calibration performance. When using two models, one model predicts the mean and the other predicts a single quantile, which can be converted into the standard deviation using the same method.

#### Uncertainty quantification evaluation

To ensure the uncertainty quantification is accurate, the calibration and sharpness of the predictive distribution should be evaluated. To measure the calibration, the (Absolute) Distribution Coverage Error ((A)DCE) is proposed for heuristic calibration calculation in distributions, based on the Prediction Interval Coverage Percentage [32] that calculates the empirical coverage of a prediction interval with upper and lower bounds $y_{U}$ and $y_{L}$ :

7 $$\begin{aligned} PICP({\textbf {y}},{\textbf {y}}_{L},{\textbf {y}}_{U})=\frac{1}{N}\sum _{i=1}^{N}I\{y_{L_i}\le y_i\le y_{U_i}\} \end{aligned}$$

Where *y* is the target value vector, *I* the indicator function, and N the amount of included data points.

We then define the coverage function *C*, that calculates the empirical coverage of estimated centered prediction intervals extracted from the predictive distribution with estimated parameters $\tilde{\sigma }$ and $\tilde{\mu }$ for a specified coverage *p*:

8 $$C({\mathbf{y}},p,\tilde{\sigma },\tilde{\mu }) = PICP\left( {{\mathbf{y}},Q\left( {\frac{{1 - p}}{2},\tilde{\sigma },\tilde{\mu }} \right),Q\left( {\frac{{1 + p}}{2},\tilde{\sigma },\tilde{\mu }} \right)} \right){\text{ }}$$

A sampling rate S is defined for the heuristic calculation of the calibration, corresponding to the step size of the percentages, which is set to 1000 in this work. To bound the absolute values of DCE and ADCE to [0, 1] and thereby, both are multiplied by 2:

9 $$\begin{aligned}&DCE(y,\tilde{\theta })=\frac{2}{S}\sum _{i=0}^{S}\left( C(y,i/S,\tilde{\sigma },\tilde{\mu })-\frac{i}{S}\right) \end{aligned}$$

10 $$\begin{aligned}&ADCE(y,\tilde{\theta })=\frac{2}{S}\sum _{i=0}^{S}\left| C(y,i/S,\tilde{\sigma },\tilde{\mu })-\frac{i}{S}\right| \end{aligned}$$

The ADCE quantifies the average calibration of the complete predictive distribution (for a more elaborate explanation of average calibration we refer to [33]). The DCE shows any calibration biases, either consistently underestimating (positive) or overestimating its coverage (negative). However, do note that the DCE can be 0 while ADCE can be 1, but not vice versa. Therefore, it is advised to always provide both.

The calibration can then be plotted in calibration plots, plotting the coverage *C* for each *p*, for further visual inspection of the calibration performance.

### Hyperparameters

#### Gradient boosting trees (GBT)

Four hyperparameters of the GBT ensemble were optimized using cross-validation: tree depth, leaf regularization, border count, and the quantile coverage *p*. The final hyperparameters of all GBT *new* sub-models were chosen to be 4, 1, 250, and 0.80, respectively. For the GBT *prev* model, they were 3, 4, 50, and 0.82, respectively. Therefore, the loss function of the *new* and *prev* sub-model responsible for the regression output was $Quantile:alpha=0.5$. For the *new* upper and lower quantile models the loss functions were $Quantile:alpha=0.90$ and $Quantile:alpha=0.10$ respectively, while they were $Quantile:alpha=0.91$ and $Quantile:alpha=0.09$ for the *prev* upper and lower quantile models.

#### Gaussian processes (GP)

For optimizing the GP, at least one feature is required to determine the kernel but a kernel is required to perform feature selection. Since the feature with the largest correlation to the concentration has a high chance of being included in the final feature set and can thus be used for kernel selection. The feature with the largest Pearson correlation to the concentration, $CL_{cr}$, was chosen to determine the most adequate kernel. The GP *prev* model used the following features (ordered in descending importance): previous concentration (MLP kernel weight variance $=$ 0.085; higher weight indicates larger importance), creatinine clearance (0.080), serum creatinin (0.056), and fluid balance (0.0078). The GP *new* model used the following features: creatinine clearance (RBF kernel lengthscale $=$ 0.95; lower weight indicates larger importance), serum creatinin (1.73), albumin (13.45), and bilirubin (152.7).

#### Multi-layer perceptron (MLP)

The developed neural network is a neural network with 3 layers, each with a width of 32 nodes and using the ReLu activation function. The models were optimized using the Tensorflow Adam optimizer with a learning rate of 0.0005, batch size of 32, and 75 epochs.

### Population PK model

A two-compartmental piperacillin PopPK model with parallel linear/Michaelis-Menten elimination was used to predict antimicrobial concentrations for model comparison [43]. In this PopPK model, the m$CL_{cr}$ (mL/min), normalized to 100mL/min, and the body weight, normalized to 70kg, were included for determining the clearance using a power function with 0.75 as an exponent. The volume of distribution had an exponent of 1. This model is described as follows:

11 $$\begin{aligned}&CL =TVCL\left( \frac{CL_{CR}}{100}\right) \left( \frac{WEIGHT}{70}\right) ^{0.75} \end{aligned}$$

12 $$\begin{aligned}&V = TVV\left( \frac{WEIGHT}{70}\right) \end{aligned}$$

13 $$\begin{aligned}&V_p = TVV_p\left( \frac{WEIGHT}{70}\right) \end{aligned}$$

14 $$\begin{aligned}&Q = TVQ\left( \frac{WEIGHT}{70}\right) ^{0.75} \end{aligned}$$

The median and 95% confidence intervals for model parameters drug clearance (*CL*), volume of the central compartment (*V*), volume of the peripheral compartment ($V_p$), and intercompartmental clearance (*Q*) were 9 (7.69–11) L/h, 6.18 (4.9–11.2) L, 11.17 (7.26–12) L, and 15.61 (12.66–23.8) L/h. The Michaelis–Menten constant ($K_m$) and the maximum elimination rate for Michaelis–Menten elimination ($V_{max}$) were estimated without population variability in the model to avoid overfitting. The population estimates for $K_m$ and $V_{max}$ were 37.09 mg/L and 353.57 mg/h, respectively.

NONMEM®(version 7.5; GloboMax LCC, Hanover, MD, CA, USA) was used to predict antimicrobial concentrations with the published PopPK model. Predictions made with the PopPK model were deterministic, i.e. without residual uncertainty.

### Concentration prediction evaluation

For *a priori* evaluation, *a priori* PopPK predictions were generated with a parameter distribution equal to the population parameter distribution of the PopPK model (i.e. Bayesian prior) and compared to the *new* ML models. For *a posteriori* evaluation, individual PK parameter estimates, as opposed to population PK parameter estimates, can be used to generate *a posteriori* predictions (the Bayesian posterior), and compared to the *prev* ML models [44–46]. All used ML models provide deterministic predictions.

PopPK and ML predictions for the GUH database were also converted into different categories to assess target attainment, required in clinical practice. The first category, subtherapeutic, contains unbound concentrations below the target attainment value of four times the minimum inhibitory concentration (MIC) of Pseudomonas aeruginosa of 16 mg/L [47]. This breakpoint, the upper limit of piperacillin susceptibility, represents a worst-case scenario for empirical dosing for when the MIC of the pathogen is not yet known. However, this can be changed when the MIC of the pathogen is known. The supratherapeutic category is based upon the toxicity risk of TZP, set at an unbound concentration of 112 mg/L [48]. The therapeutic category lies in between these two categories. Classification performance was evaluated using the precision (i.e. positive predictive value (PPV)), specificity (i.e. selectivity or true negative rate (TNR)), sensitivity (i.e. recall, hit rate, or true positive rate (TPR)), and F1-score metrics.

As only total plasma concentrations were measured, a protein binding factor of 30% was considered, resulting in a subtherapeutic threshold of 91.43 mg/L and a supratherapeutic threshold of 160 mg/L [49].

## Results

### Final features

Features were collected on the plausibility of prediction impact as judged by clinicians and by permission of collection. Adding features to include changes over time did not result in better performances and were hence not included. Features included in the different ML models after feature selection can be seen in Table 3, any features not present in the table were not in the final feature set of any model as they did not increase the performance. The GP models did not use many features as they are highly sensitive to high dimensions and therefore prefer small feature sets. The features included in every model are the creatinine clearance and the serum creatinine and prove their already known predictive capabilities. Albumin return in four of the five ML models as a feature, indicating it as important for predicting the piperacillin plasma concentrations. Weight is seen as an important indicator for the volume of distribution in the PopPK model. However, no ML model included weight, as the addition of weight in the cross-validation phase only decreased performance always preferring height over weight. As an experiment, when height was not available as a feature, the models included weight as a predictor, indicating that the models found height more informative than weight in this dataset. In the GUH dataset, the weight and the height were normally distributed with the same standard deviation and contained only a few outliers. As a result, model predictions for patients with an outlying weight could be less optimal.

**Table 3:** Features used by each model

Feature	GBT prev	GBT new	GP prev	GP new	MLP	PopPK
Albumine (g/dL)	X	X		X	X	
Bilirubine (mg/dL)	X	X		X		
Creatinine clearance (mL/min)	X	X	X	X	X	X
Fluid balance (mL/24 h)		X	X			
Height (cm)	X	X			X	
Lactate (mg/dL)	X	X				
Platelets (/mm$^{3}$)	X	X			X	
Red blood cells (/mm$^{3}$)	X				X	
Previous concentration (mg/L)	X		X		X	
Sex					X	
Hours since start treatment (h)	X					
Serum creatinine (mg/dL)	X	X	X	X	X	
Weight (kg)						X

### Visual interpretation of ML models

**Fig. 1:**
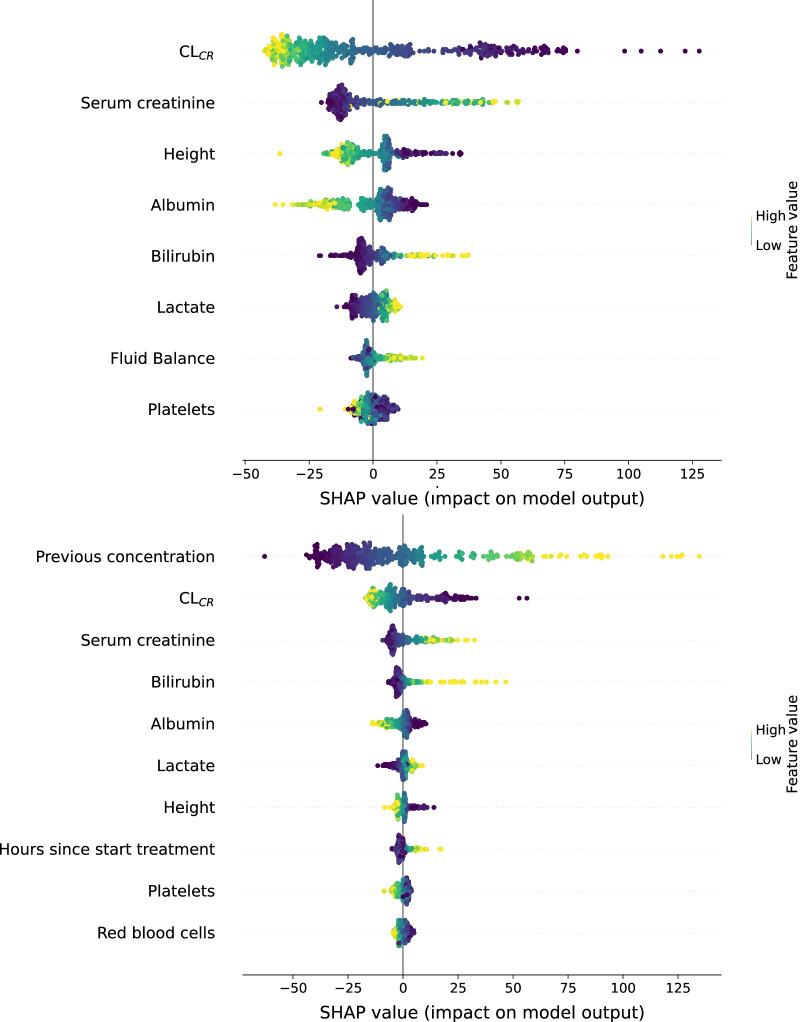
SHAP visualization for GBT *new* (top) and GBT *prev* (bottom) The SHAP values are in mg/L

Visualization of the shapely additive explanation values (SHAP) [30] for the GBT *prev* and *new* model are shown in Fig. 1. The SHAP value quantifies the impact of a feature on pushing the concentration output from the baseline prediction to the actual prediction. The baseline is considered the average of all predictions in training [30]. Both figures show the $CL_{cr}$ as the most important predictor where a low $CL_{cr}$ results in high piperacillin concentrations, in accordance with the literature.

### Evaluation

#### Concentration prediction

The concentration prediction performance on the GUH and UMCG datasets is shown in Table 4. The metrics are also calculated in the log scale as many large concentration values might skew the regression results.

**Table 4:** Evaluation performance of all considered ML and PopPK models

Model	RMSE	MAE	ME	${R^2}$	MdAPE	MdPE
*GUH evaluation: a priori*						
GBT *new*	**34.27** (**0.38**)	**21.55 **(**0.25**)	− 4.09 (**0.00**)	**0.58** (**0.57**)	**17.29**% (**3.83**%)	**0.06**% (**0.01**%)
GP *new*	37.41 (0.43)	23.54 (0.28)	2.04 (0.07)	0.50 (0.46)	21.39% (4.79%)	− 3.83% (− 0.84%)
MLP	38.56 (0.47)	27.35 (0.34)	2.58 (0.05)	0.47 (0.36)	23.09% (5.29%)	− 5.34% (− 1.26%)
PopPK	57.97 (0.64)	39.67 (0.54)	− 30.27 (− 0.45)	− 0.19 (− 0.21)	40.79% (11.60%)	38.33% (11.41%)
*GUH evaluation: a posteriori*						
GBT *prev*	**32.93 **(**0.27**)	**18.22** (**0.19**)	− 6.55 (− 0.02)	** 0.62** (**0.73**)	**12.75**% (**3.09**%)	**1.77**% (**0.43**%)
GP *prev*	34.03 (0.28)	19.41 (0.21)	**− 3.83** (**− 0.01**)	0.59 (0.71)	16.48% (3.79%)	− 3.76% (− 0.92%)
MLP	37.20 (0.36)	23.64 (0.26)	− 4.87 (− 0.03)	0.51 (0.51)	17.06% (4.14%)	0.73% (0.17%)
PopPK	49.58 (0.43)	31.28 (0.32)	4.91 (0.03)	0.14 (0.32)	26.09% (6.69%)	− 1.85% (− 0.43%)
U*MCG evaluation: a priori*						
GBT *new*	**43.92** (0.78)	38.67 (0.62)	**30.67** (0.58)	** 0.36** (− 0.12)	68.38% (12.89%)	− 68.38% (− 12.89%)
GP *new*	64.99 (0.89)	55.31 (0.74)	50.90 (0.72)	− 0.39 (− 0.45)	84.88% (15.33%)	− 84.88% (− 15.33%)
MLP	62.28 (0.85)	51.47 (0.71)	38.52 (0.63)	− 0.28 (− 0.33)	83.09% (14.97%)	− 83.09% (− 14.97%)
PopPK	50.46 (**0.67**)	**31.50** (**0.55**)	− 23.97 (**− 0.30**)	0.16 (**0.18**)	**39.84**% (**12.31**%)	**33.88** % (**9.85**%)
*UMCG evaluation: a posteriori*						
GBT *prev*	28.12 (0.57)	21.11(0.40)	15.01 (0.37)	0.68 (0.25)	37.20% (8.46%)	− 37.20% (− 8.46%)
GP *prev*	31.58 (**0.57**)	22.73 (**0.39**)	18.05 (0.35)	0.60 (**0.26**)	**25.15**% (**6.90**%)	− 25.15% (− 6.9%)
MLP	30.35 (0.64)	26.55 (0.50)	22.45 (0.47)	0.63 (0.06)	54.16% (10.48 %)	− 54.16% (− 10.48%)
PopPK	**25.89** (0.62)	**19.95** (0.45)	**2.15** (**− 0.00**)	**0.73** (0.13)	26.69% (7.31%)	**3.31**% (**0.87**%)

All RMSE, MAE, and ME values are in mg/L. The values in parenthesises are in log scale. Bold indicates the best model for that metric and case

#### Target attainment prediction

*A priori* and *a posteriori* performance of the ML models and the PopPK model on the GUH database for target attainment prediction is summarized in Table 5. This was not performed for the UMCG dataset as the number of samples was insufficient. As the MLP model was solely used to show the limited performance of a deep learning model, the MLP model was not included for target attainment prediction.

**Table 5:** GUH *a priori* classification performance of the ML and PopPK models

Model	Range	Precision	Specificity	Sensitivity	F1-score	Support
*A prior*i						
GBT *new*	Sub.	**0.88**	**0.88**	0.89	**0.88**	99
	Ther.	**0.62**	0.58	**0.69**	**0.65**	35
	Sup.	**0.67**	0.77	0.47	**0.55**	17
GP *new*	Sub.	0.88	0.88	0.85	0.87	99
	Ther.	0.53	0.47	0.60	0.56	35
	Sup.	0.56	0.58	**0.53**	0.55	17
PopPK	Sub.	0.71	0.60	**0.98**	0.82	99
	Ther.	0.50	**0.89**	0.11	0.19	35
	Sup.	0.83	**0.94**	0.29	0.43	17
*A posteriori*						
GBT *prev*	Sub.	**0.93**	**0.93**	**0.92**	**0.93**	76
	Ther.	0.63	**0.54**	**0.79**	**0.70**	24
	Sup.	**0.75**	**0.89**	0.33	0.46	9
GP *prev*	Sub.	0.92	0.92	0.92	0.92	76
	Ther.	**0.59**	0.53	0.67	0.63	24
	Sup.	0.50	0.67	0.33	0.40	9
PopPK	Sub.	0.84	0.86	0.75	0.79	76
	Ther.	0.35	0.15	0.46	0.40	24
	Sup.	0.50	0.44	** 0.56**	**0.53**	9

Subtherapeutic (Sub.): < 91.43 mg/L, Therapeutic (Ther.): $\ge$91.43 mg/L and < 160 mg/L, Supratherapeutic (Sup.): $\ge$160 mg/L. Support indicates the number of samples in that range. Bold indicates the best model for that metric and case

#### Compensation of missing creatinine clearances

The most important feature in both models is the creatinine clearance, however, not all ICUs routinely measure this covariate and instead use eGFR formulas. To evaluate these cases, all measured $CL_{cr}$ values in the test set are replaced using the weighted eGFR formula. The evaluation results of the best models, GBT *new* and GBT *prev*, on the GUH dataset for this case were (RMSE/MAE/ME/$R^2$) 40.71/27.84/− 7.24/0.41 and 34.51/19.37/− 5.75/0.58, respectively. As expected, the performance is worse. However, the models are still usable and therefore the weighted formula is an alternative for cases where the creatinine clearance cannot be measured.

### Uncertainty quantification

**Fig. 2:**
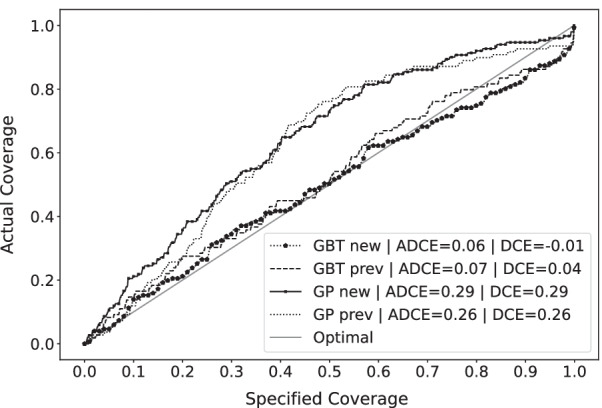
Coverage plot of all uncertainty quantification models on the GUH dataset The specified coverage is the *p* to provide the prediction intervals. The actual coverage is the measured coverage *C*

**Fig. 3:**
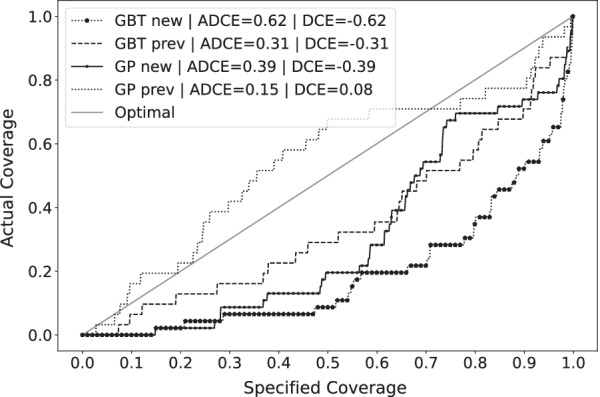
Coverage plot of the uncertainty quantification models on the UMCG dataset. The specified coverage is the *p* to provide the prediction intervals. The actual coverage is the measured coverage *C*

In Table 6, the calibration and sharpness results of both the GBT and the GP model are shown. Figures 2 and 3 shows the visualization of the calibrations for the GUH and the UMCG dataset respectively.

**Table 6:** Uncertainty quantification performance of the GBT models and the GP models

Model	ADCE	DCE	Sharpness (std) (mg/L)
*GUH evaluation: a priori*			
GBT *new*	**0.06**	**0.01**	**23.48 (11.41)**
GP *new*	0.29	0.29	41.22 (4.26)
*GUH evaluation: a posteriori*			
GBT *prev*	**0.07**	**0.04**	**17.98(8.62)**
GP *prev*	0.28	0.28	28.94 (0.86)
*UMCG evaluation: a priori*			
GBT *new*	0.62	0.62	**25.50 (13.43)**
GP *new*	**0.39**	**− 0.39**	42.75 (9.67)
*UMCG evaluation: a posteriori*			
GBT *prev*	0.31	− 0.31	**17.61 (8.02)**
GP *prev*	**0.15**	**0.08**	28.22 (0.99)

Bold indicates the best model for that metric and case

### Patient case study

One patient will be discussed in this section to illustrate what information the final ML models can provide to the clinician when predicting the plasma concentration in clinical practice. The discussed patient is a patient with a previously measured concentration and is therefore handled by the GBT *prev* model and *a posteriori* PopPK model for comparison. The features at the time of measuring the discussed observed plasma concentration of this patient are shown in Table 7 .

**Table 7:** Features of the discussed patient

Height (cm)	Serum creatinine (mg/dL)	Platelets (plt/mm^3^)	Bilirubin (mg/dL)	Lactate (mg/dL)
170	0.51	248.0	1.7	9.1

Albumin (g/L)	Hours since start treatment (h)	$CL_{Cr}$ (mL/min)	Red blood cells (/mm^3^)	
20	78	72.60	3.75

**Table 8:** A posteriori PopPK prediction

CL	V	Q	${\text {V}}_p$	${\text {K}}_m$	${\text {V}}_{max}$	Pred (mg/L)
2.36	6.01	15.30	10.90	37.10	354.0	161.0

**Fig. 4:**
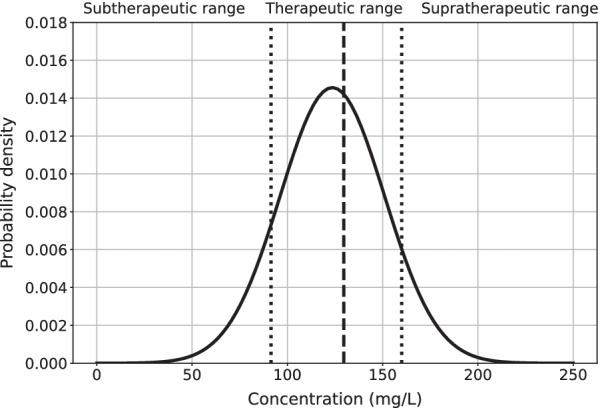
SHAP visualization for a given patient with the GBT *prev* model. The red values increase the output while the blue values decrease the output. The mentioned values are piperacillin plasma concentrations (mg/L)

**Fig. 5:**
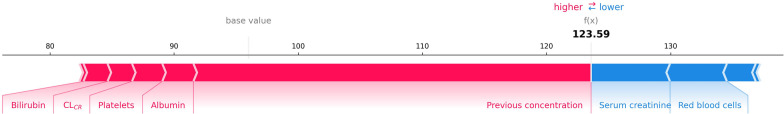
Prediction output of the first discussed patient with the GBT *prev* model. The dashed (middle) line is the observed concentration and the dotted (outer) lines indicate the therapeutic range boundaries

The patient suffered a wound infection in the lower legs with amputation of the right leg. The observed plasma concentration was 129.60 mg/L and the patient had a previous concentration of 173.40 mg/L. The GBT *prev* model predicted a piperacillin plasma concentration of 123.59 mg/L with an estimated standard deviation of 27.40 mg/L while the *a posteriori* PopPK model predicted 161.0 mg/L. The output distribution of the ML model is visualized in Fig. 4 and the estimated PopPK parameters are shown in Table 8. With this information, the patient was determined by the ML model to be in the therapeutic dosing range with 76.1% certainty, in the subtherapeutic range with 13.4% certainty, and 10.5% certainty for the supratherapeutic range. The influence of each feature is shown in Fig. 5 using the SHAP-values. Both the dose per hour and the height are not visible in the SHAP plots as their SHAP values are too small to visualize. Here we can see that the previous plasma concentration has the highest impact on the output due to its high value of 173.40 mg/L, the low serum creatinine has the second-highest impact and reduces the final prediction. All *a posteriori* PopPK model estimates were: *CL* = 2.36 L/h, *V* = 6.01 L, *Q* = 15.30 L/h, $V_p$ = 10.90 L, $K_m$ = 37.10 mg/L, and $V_{max}$ = 354.0 mg/h. The PopPK model had a noticeably low clearance while the other parameters are average values, possibly resulting in overpredicting the concentration.

## Discussion

The ML models were developed with piperacillin plasma concentrations from critically ill patients receiving a continuous TZP infusion. The model with the smallest bias and imprecision for the piperacillin concentration predictions on the GUH evaluation sets was the GBT model. The PopPK model showed better performance on the UMCG evaluation *a posteriori* set in the natural scale. In log-scale, the GP *prev* and the GBT *prev* models performed better, while this is reversed for the *a priori* UMCG test set. All models tend to lose performance for higher concentrations (i.e. the supratherapeutic range), which can be explained by the lack of data in this range.

Predicted drug concentrations are point estimates and reporting the degree of uncertainty is important for clinical decision-making [21]. To this end, we proposed the Quantile Ensemble method and compared it to a Gaussian process model to provide and evaluate reliable prediction intervals and predictive distributions. Looking at the results, the GP model’s predictive distribution calibration and sharpness are much worse than the GBT model. The size of the predictive distribution of the GBT models is around 40% smaller than the GP models while achieving much better calibration metrics. Furthermore, the GP model sharpness standard deviation is low, showing the homoscedastic nature of the model and its negative impact on uncertainty quantification. The GP model has to increase the size of the predictive distribution to account for the global uncertainty prediction, as a result, the individual uncertainty predictions have very large bounds and are the same for every patient. Subsequently, the GP model uncertainty prediction has no real clinical value as a more individualized prediction is preferred for each patient. Looking at the GUH test set ADCE and DCE values and the calibration plots, the large positive GP model DCE values indicate a large conservative uncertainty prediction. This behavior proves that the size of the distribution can be much smaller on average, further supported by the large GP model sharpness and the better GBT model performances as mentioned previously. Furthermore, we can also conclude that the GBT model still provides slightly conservative uncertainty predictions, given the small positive DCE. These results show the added value and the strength of the heteroscedastic approach of the Quantile Ensemble method, even on a small dataset, and the interpretability and strength of the ADCE and DCE metrics, even in model selection.

Together with the regression and uncertainty quantification performance, the preferred model is the GBT model. For clinical practice, Struys et al. defined the threshold for drug prediction model acceptance as $30\%$ for MdAPE and $[-20\%, 20\%]$ for MdPE on the natural concentration scale [50]. If we were to apply this to all models, then only the *a priori* PopPK model does not achieve this minimum requirement. For external evaluation, only the *a posteriori* PopPK model achieves the requirement.

ML models are often perceived as ‘black-box’ models and, when it comes to ML for the prediction of drug concentrations, it may be difficult to understand how concentration ‘X’ is predicted, and dose ‘Y’ is suggested. End-user interpretability is largely determined by the choice of specific ML techniques such as GBT, which can provide insight into the model output (‘white-box’). As shown, visualization libraries, like SHAP, may further increase the understanding of end-users and thereby lower the threshold for ML adaptation in clinical practice.

This study is limited by only using plasma concentrations from ICU patients receiving continuous infusion TZP. Therefore, the findings of this study cannot yet be extrapolated to other antimicrobial drugs or alternative modes of infusion instead of continuous infusion. Additionally, the used piperacillin concentrations are total plasma concentrations and not tissue concentrations. Only the unbound drug fraction at the site of infection can exert its antimicrobial effect. Furthermore, tissue perfusion of critically ill patients is unpredictable, therapeutic plasma concentrations may not necessarily predict therapeutic tissue concentrations, however, attaining sufficiently high plasma concentrations is required for achieving therapeutic tissue concentrations [5].

Renal replacement therapy (RRT) patients were not included. Therefore, there was no training on these patients and the performance will likely be worse when using the model on RRT patients. As a backup, the weighted CG and MDRD formula can be used when the patient is on RRT, however, this solution is not validated. The main challenge of modeling RRT patients is creating a surrogate for the $CL_{cr}$ that can be used as a feature in the model.

The dataset for external model evaluation (UMCG dataset) is small. Therefore, reliably extrapolating these results to other hospitals is not yet possible. As the UMCG patients received lower doses and the ML models assume the GUH dosing scheme, they, therefore, overestimate the external validation concentrations which is visible in the high ME. If we compensate for this bias, i.e. by subtracting the ME from the predictions, the $R^2$ (log scale) becomes 0.77 (0.56) and the RMSE becomes 23.78 (0.44) for the GBT *prev* model and 0.67 (0.50) and 31.44 (0.52) for the *new* model, further proving this is a bias introduced due to the different dosing as this model then outperforms the PopPK *a posteriori* model. The MdAPE and MdPE of the compensated *prev* GBT model also reduces to $17.92\%$ and $-1.55\%$, making it an acceptable model by the criteria of Struys et al. [50]. The same conclusion can be made for the *new* GBT model (MdAPE $=$ $20.69\%$, MdPE $=$ $0.39\%$). As a result, the model shows generalization capabilities if adjusting for a different dosing range is possible, however, these bias-compensated models should then be further validated on a new dataset before acceptance. As the external dataset was too small, this was not possible.

The dosing bias also explains the worse uncertainty quantification performance on the UMCG dataset, as the uncertainty quantification method is not robust against this kind of data bias, resulting in a highly negative biased calibration (i.e., the predictive distribution is not wide enough). If we also calculate the calibration error on the compensated results, the ADCE and DCE becomes 0.13 and 0.11 for the GBT *new* model, and 0.15 and 0.14 for the *prev* model. Interestingly, the positive DCE on the compensated results proves that the predictive distribution is even slightly too wide for the external evaluation proving the generalization capabilities of the Quantile Ensemble method.

Further work should try to adjust for different dosing regimens. Since the GUH only contained a single dosing scheme, training on different dosings was not possible. This will also enable dosing suggestions when the estimated plasma concentration is not in the therapeutic dosing range.

Lastly, the ML models do not currently take time into account. Therefore, calculating time above the MIC (%fT > MIC), the pharmacokinetic/pharmacodynamic index for beta-lactam antimicrobials, is not entirely possible with the current ML model. However, the models still do indicate the plasma concentration which can be used for initial dose optimization. True dose optimization based on PK/PD target attainment is an area needing further research and can combine the PopPK modeling techniques with the predictive power of ML [5].

Overall, the proposed models demonstrate that this can be considered as an alternative strategy to guide antibiotic therapy, in addition to PopPK methods, by predicting plasma antibiotic concentration while also providing uncertainty estimation. As a result, this opens the path to incorporating machine learning models in decision support systems for more individualized and targeted antibiotic therapy. Furthermore, both the uncertainty framework and the ACDE and DCE metrics can be applied to many more use cases by following the same approach to enable uncertainty quantification and uncertainty evaluation. The presented piperacillin models presented in the paper are based on retrospective analysis. The next step, in future work, is performing a prospective study together with dose suggestion. The final aim is deployment in clinical practice in the intensive care unit by integration it with the electronic health records for real-time concentration predictions.

## Conclusion

Our results show that ML models can consistently estimate piperacillin concentrations with high predictive accuracy, especially when no previous concentration is available, and special emphasis was placed on the interpretability of ML model output using SHAP visualization. Furthermore, the method of generating a predictive distribution using the Quantile Ensemble model can be translated into many other regression problems using any ML model and optimizing a quantile loss function. Additionally, this work also proposed the (Absolute) Distribution Coverage Error, an interpretable uncertainty quantification evaluation metric, usable for any distribution-based uncertainty quantification method.

As such, incorporating ML models in therapeutic drug monitoring programs is definitely promising. Furthermore, these results create a model that is ready to be validated in clinical practice, or at least, in the locally developed hospital.

## Data Availability

The data that supports the findings of this study are available from Ghent University Hospital but restrictions apply to the availability of these data, which were used under license for the current study, and so are not publicly available. The data is however available from the authors upon reasonable request and with permission of Ghent University Hospital Ethics Committee. The code supporting the conclusions of this article is available in the predict-idlab/REACT repository, https://github.com/predict-idlab/REACT.

## Abbreviations

ICU: Intensive care unit
TZP: Piperacillin/tazobactam
TDM: Therapeutic drug monitoring
PK: Pharmacokinetic
PopPK: Population pharmacokinetic
ML: Machine learning
GUH: Ghent University Hospital
RRT: Renal replacement therapy
SOFA: Sequential Organ Failure Assessment
APACHE II: Acute physiology and chronic health evaluation
Clcr: Creatinine clearance
mCLcr: measured creatinine clearance
eGFR: Estimated glomerular filtration rate
UMCG: University Medical Centre of Groningen
GP: Gaussian process
MLP: Multiplayer perceptron
GBT: Gradient boosting tree
IQR: Interquartile range
CV: Cross-validation
ME: Mean error
MAE: Mean absolute error
RMSE: Root mean square error
R: Coefficient of determination
MdAPE: Median absolute percentage error
MdPE: Median percentage error
ADCE: Absolute distribution coverage error
DCE: Distribution coverage error
PICP: Prediction interval coverage percentage
MIC: Minimum inhibitory concentration
SHAP: Shapely additive explanation values

## References

[1] Neidell MJ, Cohen B, Furuya Y, Hill J, Jeon CY, Glied S, Larson EL (2012). Costs of Healthcare- and community-associated infections with antimicrobial-resistant versus antimicrobial-susceptible organisms. Clin Infect Dis.

[2] Sakr Y, Jaschinski U, Wittebole X, Szakmany T, Lipman J, Namendys-Silva SA, Martin-Loeches I, Leone M, Lupu M-N, Vincent J-L (2018). Sepsis in intensive care unit patients: worldwide data from the intensive care over nations audit. Open Forum Infect Dis.

[3] Roberts JA, Roger C, De Waele JJ (2019). Personalized antibiotic dosing for the critically ill. Intensive Care Med.

[4] De Waele JJ, Akova M, Antonelli M, Canton R, Carlet J, De Backer D, Dimopoulos G, Garnacho-Montero J, Kesecioglu J, Lipman J, Mer M, Paiva J-A, Poljak M, Roberts JA, Rodriguez Bano J, Timsit J-F, Zahar J-R, Bassetti M (2018). Antimicrobial resistance and antibiotic stewardship programs in the ICU: insistence and persistence in the fight against resistance. A position statement from ESICM/ESCMID/WAAAR round table on multi-drug resistance. Intensive Care Med.

[5] Roberts JA, Abdul-Aziz MH, Lipman J, Mouton JW, Vinks AA, Felton TW, Hope WW, Farkas A, Neely MN, Schentag JJ, Drusano G, Frey OR, Theuretzbacher U, Kuti JL (2014). International society of anti-infective pharmacology and the pharmacokinetics and pharmacodynamics study group of the European Society of clinical microbiology and infectious diseases: individualised antibiotic dosing for patients who are critically ill: challenges and potential solutions. Lancet Infect Dis.

[6] Tängdén T, Ramos Martìn V, Felton TW, Nielsen EI, Marchand S, Brüggemann RJ, Bulitta JB, Bassetti M, Theuretzbacher U, Tsuji BT, Wareham DW, Friberg LE, De Waele JJ, Tam VH, Roberts JA (2017). The role of infection models and PK/PD modelling for optimising care of critically ill patients with severe infections. Intensive Care Med.

[7] Tabah A, De Waele J, Lipman J, Zahar JR, Cotta MO, Barton G, Timsit J-F, Roberts JA (2015). The ADMIN-ICU survey: a survey on antimicrobial dosing and monitoring in ICUs. J Antimicrob Chemother.

[8] Roberts JA, Kumar A, Lipman J (2017). Right dose, right now: customized drug dosing in the critically ill. Crit Care Med.

[9] Roberts JA, Abdul-Aziz M-H, Davis JS, Dulhunty JM, Cotta MO, Myburgh J, Bellomo R, Lipman J (2016). Continuous versus Intermittent beta-Lactam infusion in severe sepsis. A meta-analysis of individual patient data from randomized trials. Am J Respir Crit Care Med.

[10] Richter DC, Frey O, Röhr A, Roberts JA, Köberer A, Fuchs T, Papadimas N, Heinzel-Gutenbrunner M, Brenner T, Lichtenstern C, Weigand MA, Brinkmann A (2019). Therapeutic drug monitoring-guided continuous infusion of piperacillin/tazobactam significantly improves pharmacokinetic target attainment in critically ill patients: a retrospective analysis of four years of clinical experience. Infection.

[11] Wong G, Brinkman A, Benefield RJ, Carlier M, De Waele JJ, El Helali N, Frey O, Harbarth S, Huttner A, McWhinney B, Misset B, Pea F, Preisenberger J, Roberts MS, Robertson TA, Roehr A, Sime FB, Taccone FS, Ungerer JPJ, Lipman J, Roberts JA (2014). An international, multicentre survey of beta-lactam antibiotic therapeutic drug monitoring practice in intensive care units. J Antimicrob Chemother.

[12] Carlier M, Stove V, Wallis SC, De Waele JJ, Verstraete AG, Lipman J, Roberts JA (2015). Assays for therapeutic drug monitoring of beta-lactam antibiotics: a structured review. Int J Antimicrob Agents.

[13] Sherwin CMT, Kiang TKL, Spigarelli MG, Ensom MHH (2012). Fundamentals of population pharmacokinetic modelling: validation methods. Clin Pharmacokinet.

[14] Gonçalves-Pereira J, Póvoa P (2011). Antibiotics in critically ill patients: a systematic review of the pharmacokinetics of beta-lactams. Crit Care (Lond, Engl).

[15] Bonate PL. Pharmacokinetic–pharmacodynamic modeling and simulation, 2nd edn. Springer. 10.1007/978-1-4419-9485-1. https://www.springer.com/gp/book/9781441994844 (2011). Accessed 15 June 2021.

[16] Wong G, Farkas A, Sussman R, Daroczi G, Hope WW, Lipman J, Roberts JA (2015). Comparison of the accuracy and precision of pharmacokinetic equations to predict free meropenem concentrations in critically ill patients. Antimicrob Agents Chemother.

[17] Dhaese SAM, Farkas A, Colin P, Lipman J, Stove V, Verstraete AG, Roberts JA, De Waele JJ (2019). Population pharmacokinetics and evaluation of the predictive performance of pharmacokinetic models in critically ill patients receiving continuous infusion meropenem: a comparison of eight pharmacokinetic models. J Antimicrob Chemother.

[18] Colin PJ, Allegaert K, Thomson AH, Touw DJ, Dolton M, de Hoog M, Roberts JA, Adane ED, Yamamoto M, Santos-Buelga D, Martín-Suarez A, Simon N, Taccone FS, Lo Y-L, Barcia E, Struys MMRF, Eleveld DJ (2019). Vancomycin Pharmacokinetics throughout life: results from a pooled population analysis and evaluation of current dosing recommendations. Clin Pharmacokinet.

[19] De Corte T, Elbers P, De Waele J (2021). The future of antimicrobial dosing in the ICU: an opportunity for data science. Intensive Care Med.

[20] Lancet T (2017). Artificial intelligence in health care: within touching distance. The Lancet.

[21] Kümmel A, Bonate P.L, Dingemanse J, Krause A (2018). Confidence and prediction intervals for pharmacometric models. CPT: Pharmacomet Syst Pharmacol.

[22] Tang J, Liu R, Zhang Y-L, Liu M-Z, Hu Y-F, Shao M-J, Zhu L-J, Xin H-W, Feng G-W, Shang W-J, Meng X-G, Zhang L-R, Ming Y-Z, Zhang W (2017). Application of machine-learning models to predict tacrolimus stable dose in renal transplant recipients. Sci Rep.

[23] Liu R, Li X, Zhang W, Zhou H-H (2015). Comparison of nine statistical model based warfarin pharmacogenetic dosing algorithms using the racially diverse international warfarin pharmacogenetic consortium cohort database. PLOS ONE.

[24] Poynton MR, Choi BM, Kim YM, Park IS, Noh GJ, Hong SO, Boo YK, Kang SH (2009). Machine learning methods applied to pharmacokinetic modelling of remifentanil in healthy volunteers: a multi-method comparison. J Int Med Res.

[25] Guo W, Yu Z, Gao Y, Lan X, Zang Y, Yu P, Wang Z, Sun W, Hao X, Gao F (2021). A machine learning model to predict risperidone active moiety concentration based on initial therapeutic drug monitoring. Front Psychiatry.

[26] Shakeel D, Mir S.A (2020). Personalized drug concentration predictions with machine learning: an exploratory study. Int J Basic Clin Pharmacol.

[27] Mo X, Chen X, Wang X, Zhong X, Liang H, Wei Y, Deng H, Hu R, Zhang T, Chen Y, Gao X, Huang M, Li J (2022). Prediction of tacrolimus dose/weight-adjusted trough concentration in pediatric refractory nephrotic syndrome: a machine learning approach. Pharmacogenomics Personal Med.

[28] Ma P, Liu R, Gu W, Dai Q, Gan Y, Cen J, Shang S, Liu F, Chen Y (2022). Construction and interpretation of prediction model of teicoplanin trough concentration via machine learning. Front Med.

[29] Brier ME, Zurada JM, Aronoff GR (1995). Neural network predicted peak and trough gentamicin concentrations. Pharm Res.

[30] Lundberg S, Lee S-I. A Unified approach to interpreting model predictions. arXiv:1705.07874 [cs, stat] (2017) (2017). Accessed 15 June 2021.

[31] Lee S, Song M, Han J, Lee D, Kim B-H (2022). Application of machine learning classification to improve the performance of vancomycin therapeutic drug monitoring. Pharmaceutics..

[32] Pearce T, Brintrup A, Zaki M, Neely A. High-Quality Prediction Intervals for deep learning: a distribution-free, ensembled approach. In: International conference on machine learning, pp. 4075–4084. PML. ISSN: 2640–3498. http://proceedings.mlr.press/v80/pearce18a.html. (2018). Accessed 04 Mar 2021.

[33] Zhao S, Ma T, Ermon S. Individual calibration with randomized forecasting. arXiv:2006.10288 [cs, stat] (2020). Accessed 04 Mar 2021.

[34] Vincent JL, Moreno R, Takala J, Willatts S, De Mendonça A, Bruining H, Reinhart CK, Suter PM, Thijs LG (1996). The SOFA (sepsis-related organ failure assessment) score to describe organ dysfunction/failure. Intensive Care Med.

[35] Levey A.S, Stevens L.A, Schmid C.H, Zhang Y.L, Castro A.F, Feldman H.I, Kusek J.W, Eggers P, Van Lente F, Greene T, Coresh J (2009). A new equation to estimate glomerular filtration rate. Ann Intern Med.

[36] Carlier M, Stove V, De Waele JJ, Verstraete AG (2015). Ultrafast quantification of beta-lactam antibiotics in human plasma using UPLC-MS/MS. J Chromatogr B Anal Technol Biomed Life Sci.

[37] Aardema H, Nannan Panday P, Wessels M, van Hateren K, Dieperink W, Kosterink J.G.W, Alffenaar J.-W, Zijlstra J.G (2017). Target attainment with continuous dosing of piperacillin/tazobactam in critical illness: a prospective observational study. Int J Antimicrob Agents.

[38] Osborne J (2013). Best practices in data cleaning: a complete guide to everything you need to do before and after collecting your data.

[39] Levey AS, Coresh J, Greene T, Stevens LA, Zhang YL, Hendriksen S, Kusek JW, Van Lente F (2006). Chronic kidney disease epidemiology collaboration: using standardized serum creatinine values in the modification of diet in renal disease study equation for estimating glomerular filtration rate. Ann Intern Med.

[40] Cockcroft DW, Gault MH (1976). Prediction of creatinine clearance from serum creatinine. Nephron.

[41] Verhaeghe J, Van Der Donckt J, Ongenae F, Van Hoecke S. Powershap. A power-full shapley feature selection method. arXiv (2022). 10.48550/ARXIV.2206.08394. arXiv:2206.08394

[42] Bzdok D, Altman N, Krzywinski M (2018). Statistics versus machine learning. Nat Methods.

[43] Dhaese S.a.M, Colin P, Willems H, Heffernan A, Gadeyne B, Van Vooren S, Depuydt P, Hoste E, Stove V, Verstraete AG, Lipman J, Roberts JA, De Waele JJ (2019). Saturable elimination of piperacillin in critically ill patients: implications for continuous infusion. Int J Antimicrob Agents.

[44] Zwart TC, Moes DJAR, van der Boog PJM, van Erp NP, de Fijter JW, Guchelaar H-J, Keizer RJ, Ter Heine R (2021). Model-informed precision dosing of everolimus: external validation in adult renal transplant recipients. Clin Pharmacokinet.

[45] Colin PJ, Eleveld DJ, Hart A, Thomson AH (2021). Do vancomycin pharmacokinetics differ between obese and non-obese patients? Comparison of a general-purpose and four obesity-specific pharmacokinetic models. Ther Drug Monit.

[46] Broeker A, Nardecchia M, Klinker KP, Derendorf H, Day RO, Marriott DJ, Carland JE, Stocker SL, Wicha SG (2019). Towards precision dosing of vancomycin: a systematic evaluation of pharmacometric models for Bayesian forecasting. Clin Microbiol Infect: Off Publ Eur Soc Clin Microbiol Infect Dis.

[47] EUCAST: EUCAST. https://eucast.org/. Accessed 15 June 2021.

[48] Quinton M-C, Bodeau S, Kontar L, Zerbib Y, Maizel J, Slama M, Masmoudi K, Lemaire-Hurtel A-S, Bennis Y (2017). Neurotoxic concentration of piperacillin during continuous infusion in critically ill patients. Antimicrob Agents Chemother.

[49] Hayashi Y, Roberts JA, Paterson DL, Lipman J (2010). Pharmacokinetic evaluation of piperacillin–tazobactam. Expert Opin Drug Metab Toxicol.

[50] Struys M, Absalom A, Shafer SL. Intravenous drug delivery devices. In: Miller’s anesthesia, 9th edn. Elsevier; 2019.

